# Supplementary material for: Spectrum of urolith composition among a multi-ethnic population at the Aga Khan hospital, Dar-es-Salaam, Tanzania
Source: PLoS One. 2025 Aug 6;20(8):e0329564. doi: 10.1371/journal.pone.0329564 (PMC12327614; doi:10.1371/journal.pone.0329564)
Supplement: S1. Appendix — (DOCX) [file pone.0329564.s001.docx]

**Appendix**

Data collection tool

1. Serial No. ____
2. Medical Records Number _____________
3. Age ______ years
4. Sex
5. Male
6. Female
7. Place of Residence __________________
8. Nationality _________________
9. Clinical Presentation
10. Flank pain
11. Hematuria
12. Fever
13. Nausea
14. Vomiting
15. Other _____________________
16. History of OR current Urinary tract infection
17. Yes
18. No
19. Comorbidities _____________________________________
20. Mode of Diagnosis
21. Computed Tomography (CT) scan
22. X-ray
23. Ultrasound
24. Anatomical location of urolith
25. Renal
26. Pelvi-ureteric junction
27. Ureter
28. Vesicoureteric junction
29. Bladder
30. Mode of extraction of Urolith
31. Ureteroscopy (URS) + Laser Lithotripsy
32. URS + Lithoclast
33. Percutaneous Nephrolithomy (PCNL)
34. Spontaneous Passage
35. Urolith Composition ____________________________
36. Method of stone analysis _________________________
