# Supplementary material for: Spectrum of urolith composition among a multi-ethnic population at the Aga Khan hospital, Dar-es-Salaam, Tanzania
Source: PLoS One. 2025 Aug 6;20(8):e0329564. doi: 10.1371/journal.pone.0329564 (PMC12327614; doi:10.1371/journal.pone.0329564)
Supplement: Attachment — Submitted filename: Reviewer report to PLOS ONE editor.pdf [file pone.0329564.s002.pdf]

Dear Editor,

Firstly, I want to express my appreciation for the opportunity to review this paper. I have completed the review of the manuscript titled **" SPECTRUM OF UROLITH COMPOSITION AMONG A MULTI-ETHNIC POPULATION AT THE AGA KHAN HOSPITAL, DAR-ES-SALAAM, TANZANIA"** and I would like to provide my feedback and recommendation.

The study presented in the manuscript makes a valuable contribution to the field. The authors have conducted a thorough investigation and have addressed the research question with appropriate methodology and analysis. The data analysis is sound, and the findings are well-supported by the provided evidence.

Based on my review, I am pleased to recommend the acceptance of this manuscript for publication in the PLOS ONE Journal.

While I have identified a few minor suggestions for improvement, they do not detract from the overall quality and impact of the study. I have communicated these suggestions directly to the authors in my review, and I am confident that they will address them adequately in the final version.

Thank you for considering my recommendation. Please do not hesitate to reach out to me if you require any further clarification or additional information.

Sincerely,

Dr. Fatma Mohamed Abd El-Mordy (Ph.D)
Department of Pharmacognosy and Medicinal Plants,
Faculty of Pharmacy (Girls),
Al-Azhar University.
Fatmamohammed.pharmg@azhar.edu.eg

Comments to the authors:

- In the manuscript title do you mean AGA KHAN HOSPITAL Please check the spelling all over the manuscript.
- In the introduction “ Since then, there have been few studies on urinary tract stones in Tanzania and even among those none has been done to ascertain the composition of urinary tract calculi in our region..” Please cite the references of those few studies if possible.
