# Supplementary material for: Spectrum of urolith composition among a multi-ethnic population at the Aga Khan hospital, Dar-es-Salaam, Tanzania
Source: PLoS One. 2025 Aug 6;20(8):e0329564. doi: 10.1371/journal.pone.0329564 (PMC12327614; doi:10.1371/journal.pone.0329564)
Supplement: Attachment — Submitted filename: Response to Reviewers.docx [file pone.0329564.s004.docx]

Manuscript PONE-D-24-31701

To the Academic editor and reviewers.

Thank you for giving us the opportunity to submit a revised draft of the manuscript “Spectrum of urolith composition among a multi-ethnic population at the Aga Khan Hospital, Dar-es-Salaam, Tanzania. ’’ to PLOS ONE . We appreciate the time and effort that you and the reviewers dedicated to providing feedback on our manuscript. Please find our revisions and response to the reviewers’ comments in the table below.

| **S/N** | **Comment** | **Action taken** |
| --- | --- | --- |
| Journal requirements | | |
| 1. | Please ensure that your manuscript meets PLOS ONE's style requirements, including those for file naming. | Revisions have been made throughout the document to meet PLOS ONE’s style requirements also file names have been revised to meet PLOS ONE’s file naming requirements |
| 2. | We note that your Data Availability Statement is currently as follows: “All relevant data are within the manuscript and in Supporting Information files.” Please confirm at this time whether or not your submission contains all raw data required to replicate the results of your study. Authors must share the “minimal data set” for their submission. PLOS defines the minimal data set to consist of the data required to replicate all study findings reported in the article, as well as related metadata and methods (https://journals.plos.org/plosone/s/data-availability#loc-minimaldata-set-definition). For example, authors should submit the following data: - The values behind the means, standard deviations and other measures reported; - The values used to build graphs; - The points extracted from images for analysis. Authors do not need to submit their entire data set if only a portion of the data was used in the reported study. If your submission does not contain these data, please either upload them as Supporting Information files or deposit them to a stable, public repository and provide us with the relevant URLs, DOIs, or accession numbers. For a list of recommended repositories, please see https://journals.plos.org/plosone/s/recommended-repositories. If there are ethical or legal restrictions on sharing a de-identified data set, please explain them in detail (e.g., data contain potentially sensitive information, data are owned by a third-party organization, etc.) and who has imposed them (e.g., an ethics committee). Please also provide contact information for a data access committee, ethics committee, or other institutional body to which data requests may be sent. If data are owned by a third party, please indicate how others may request data access. | The data belongs to the Aga Khan University (a third party). Data may be available on reasonable request from the Aga Khan university-Dar es Salaam campus’ research administrator. Email address for correspondence has been provided in the updated Data availability statement. |
| 3. | Please ensure that you refer to Figures 1-4 in your text as, if accepted, production will need this reference to link the reader to the figure. | All figures have been referenced in the text and the figure captions have been revised. |
| 4. | We note you have included a table to which you do not refer in the text of your manuscript. Please ensure that you refer to Table 1-6 in your text; if accepted, production will need this reference to link the reader to the Table. | All tables have been referenced in the text and their captions have been revised |
| 5. | Please include the captions for supporting information files at the end of the manuscript and update any in-text citations to match accordingly.  Please include captions for your Supporting Information files at the end of your manuscript, and update any in-text citations to match accordingly. Please see our Supporting Information guidelines for more information: http://journals.plos.org/plosone/s/supporting-information. | Caption for supporting information has been added |
| 6. | Please review your reference list to ensure that it is complete and correct. If you have cited papers that have been retracted, please include the rationale for doing so in the manuscript text, or remove these references and replace them with relevant current references. Any changes to the reference list should be mentioned in the rebuttal letter that accompanies your revised manuscript. If you need to cite a retracted article, indicate the article’s retracted status in the References list and also include a citation and full reference for the retraction notice. | Reference list has been checked and updated |
| Reviewer #1’s comments | | |
| 7 | In the manuscript title do you mean Aga Khan Hospital. Please check spelling all over the manuscript. | Thank you for the correction. Yes, I meant the Aga Khan Hospital. Spelling has been corrected form ‘hopital’ to hospital, and the spelling checked throughout the document.  . |
| 8 | In the introduction, “since then there have been few studies on urinary tract stones in Tanzania and even among those none have been done to ascertain the composition of urinary tract calculi in our region”. Please cite the references of those few studies if possible. | References for these studies have been cited. |
| Reviewer #2’s comments | | |
| 9 | The topic of the study is not novel and many factors should be studied in detailed manner including family history of chronic kidney disease special dietary habits, special medications etc. | The main objective of our study was to describe the compositions of urinary tract stones in our region. We also studied a larger number of patients compared to other studies that had been previously done in our setting.  Being a retrospective study obtaining data on the mentioned factors was not possible due to inadequate information from the patients’ medical records, and this was addressed by being included as a limitation to our study. |

Best regards,

Mapendo Providence

Corresponding author
