# Supplementary material for: Spectrum of urolith composition among a multi-ethnic population at the Aga Khan hospital, Dar-es-Salaam, Tanzania
Source: PLoS One. 2025 Aug 6;20(8):e0329564. doi: 10.1371/journal.pone.0329564 (PMC12327614; doi:10.1371/journal.pone.0329564)
Supplement: Attachment — Submitted filename: Response_to_Reviewers_auresp_2.docx [file pone.0329564.s005.docx]

Manuscript PONE-D-24-31701R1

To the Academic editor and reviewers.

Thank you for giving us the opportunity to submit a revised draft of the manuscript “Spectrum of urolith composition among a multi-ethnic population at the Aga Khan Hospital, Dar-es-Salaam, Tanzania. ’’ to PLOS ONE . We appreciate the time and effort that you and the reviewers dedicated to providing feedback on our manuscript. Please find our revisions and response to the reviewers’ comments in the table below.

| **S/N** | **Comment** | **Action taken** |
| --- | --- | --- |
| **Journal requirements.** | | |
| 1. | Please review your reference list to ensure that it is complete and correct. If you have cited papers that have been retracted, please include the rationale for doing so in the manuscript text, or remove these references and replace them with relevant current references. Any changes to the reference list should be mentioned in the rebuttal letter that accompanies your revised manuscript. If you need to cite a retracted article, indicate the article’s retracted status in the References list and also include a citation and full reference for the retraction notice. | The reference list has been reviewed.  One reference by Jung et al was removed from the reference list after revisions were made to the discussion section of the manuscript and paragraphs in which it was cited removed, these revisions in the discussion section were as per the academic editor’s comments to remove general information from this section that is similar to that already mentioned in the introduction section.  Below is the full citation for the omitted reference:  Jung H, Andonian S, Assimos D, Averch T, Geavlete P, Kohjimoto Y, et al. Urolithiasis: evaluation, dietary factors, and medical management: an update of the 2014 SIU-ICUD international consultation on stone disease. World journal of urology. 2017;35(9):1331-40. |
| **Academic editor’s comments.** | | |
| 2. | “Urolith” is not a commonly used term. Therefore I recommend that the authors use “stone disease”, “kidney stone” or something similar according to the context of the sentence in which this word is used. | We have reduced use of the word “urolith” within most of the manuscript and have replaced it with either the words “urinary tract stones”, “stones”, “urinary calculi” or “calculi” depending on the sentence in which it was used. We have also added a definition of the term urolith within the introduction section.  We agree that “urolith” is not a commonly used term however there exists a number of literature which use the word urolith to describe urinary tract stones as such we request to retain some use of the word “urolith” especially within the title. |
| 3. | There are numerous spelling and punctuation errors to be corrected: for example, leaving a space before the parenthesis or a comma, starting with capital words for some words, such as Ultrasound, Computed Tomography, or writing with small letters instead of capital letters, such as American urological association, European urological association, etc. Please correct these kind of errors. | Spelling and punctuation errors have been corrected throughout the manuscript. |
| 4. | Please start the Discussion section with a summary of your results. Please also avoid mentioning the same general information that has already been mentioned in the Introduction section. | The discussion section has been revised. It has been started with a summary of the results and general information already mentioned in the introduction section has been removed. |
| 5. | Please give the ethics committee approval date and number. | Ethics committee approval date and number have been provided in the methods section of the manuscript. They have also been added to the ethical statement section of the submission process. |
| 6. | Please place the tables and figures (along with their legends) separately at the end of the manuscript. Placing them in the main text makes the reading of the manuscript broken and more difficult. | Figure captions and tables were placed within the manuscript as this was in accordance to PLOS One’s submission guidelines.  Source: [Submission Guidelines \| PLOS One](https://journals.plos.org/plosone/s/submission-guidelines)  The guidelines state that “Figure captions must be inserted in the text of the manuscript, immediately following the paragraph in which the figure is first cited (read order). Do not include captions as part of the figure files themselves or submit them in a separate document”. Regarding tables they state, “Place each table in your manuscript file directly after the paragraph in which it is first cited (read order). Do not submit your tables in separate files.”  Kindly let us know if we did not understand and follow these guidelines correctly so we can rectify accordingly. |
| 7. | Please leave a space between the numbers (n) and the percentages (%) in the tables. | A space has been placed between the numbers and the percentages in all the tables in the manuscript. |
| 8. | It might be better to reorganize the stone types in Table 4, as calcium oxalate + combines with other types can be merged, at least the ones other than calcium oxalate + calcium phosphate and calcium oxalate + uric acid, as other mixed types are less common. | As the main objective of our study was to describe the whole spectrum of compositions of the urinary tract stones found in our setting, we kindly request to maintain the categories of compositions of urinary tract stones in table 4, so as to fully describe the variety of stones found in our study. |
| Reviewer #3’s comments | | |
| 9. | The revised manuscript was good written and discussed. All comments of reviewers were addressed in the manuscript. | N/A |

Best regards,

Mapendo Providence

Corresponding author
